# Lung metastases treated with stereotactic body radiotherapy: the RSSearch® patient Registry’s experience

**DOI:** 10.1186/s13014-017-0773-4

**Published:** 2017-02-01

**Authors:** Anthony Ricco, Joanne Davis, William Rate, Jun Yang, David Perry, John Pablo, David D‘Ambrosio, Sanjeev Sharma, Srinath Sundararaman, James Kolker, Kimberly M. Creach, Rachelle Lanciano

**Affiliations:** 1 Crozer-Keystone Health System, Philadelphia CyberKnife, Havertown, PA USA; 2 The Radiosurgical Society, Sunnyvale, CA USA; 3 MedStar Franklin Square Medical Center, Baltimore, MD USA; 4 St. Joseph’s/Chandler Savannah Hospital, Savannah, GA USA; 5 East Coast Radiation Oncology Associates, Toms River, NJ USA; 6 St. Mary’s Medical Center, Huntington, WV USA; 7 Memorial Healthcare System, Hollywood, FL USA; 8 Pennsylvania Hospital of University of Pennsylvania, Philadelphia, PA USA; 9 Mercy Health, Springfield, MO USA

## Abstract

**Objectives:**

To report overall survival and local control for patients identified in the RSSearch® Patient Registry with metastatic cancer to the lung treated with SBRT.

**Methods:**

Seven hundred two patients were identified with lung metastases in the RSSearch® Registry. Of these patients, 577 patients had SBRT dose and fractionation information available. Patients were excluded if they received prior surgery, radiation, or radiofrequency ablation to the SBRT treated area. Between April 2004-July 2015, 447 patients treated with SBRT at 30 academic and community-based centers were evaluable for overall survival (OS). Three hundred four patients with 327 lesions were evaluable for local control (LC). All doses were converted to Monte Carlo equivalents and subsequent BED Gy_10_ for dose response analysis.

**Results:**

Median age was 69 years (range, 18–93 years). Median Karnofsky performance status (KPS) was 90 (range 25/75% 80–100). 49.2% of patients had prior systemic therapy. Median metastasis volume was 10.58 cc (range 25/75% 3.7–25.54 cc). Site of primary tumor included colorectal (25.7%), lung (16.6%), head and neck (11.4%), breast (9.2%), kidney (8.1%), skin (6.5%) and other (22.1%). Median dose was 50 Gy (range 25/75% 48–54) delivered in 3 fractions (range 25/75% 3–5) with a median BED of 100Gy_10_ (range 25/75% 81–136).

Median OS for the entire group was 26 months, with actuarial 1-, 3-, and 5-year OS of 74.1%, 33.3, and 21.8%, respectively. Patients with head and neck and breast cancers had longer median OS of 37 and 32 months respectively, compared to colorectal (30 months) and lung (26 months) which corresponded to 3-year actuarial OS of 51.8 and 47.9% for head and neck and breast respectively, compared to 35.8% for colorectal and 31.2% for lung.

The median LC for all patients was 53 months, with actuarial 1-, 3-, and 5-year LC rates of 80.4, 58.9, and 46.3%, respectively. There was no difference in LC by primary histologic type (*p* = 0.49). Improved LC was observed for lung metastases that received SBRT doses of BED ≥100Gy_10_ with 3-year LC rate of 77.1% compared to 45% for lung metastases treated with BED < 100Gy_10_ (*p* = 0.01). Smaller tumor volumes (<11 cc) had improved LC compared to tumor volumes > 11 cc. (*p* = 0.005) Two-year LC rates for tumor volumes < 11 cc, 11–27 cc and > 27 cc were 72.9, 64.2 and 45.6%, respectively. This correlated with improved OS with 2-year OS rates of 62.4, 60.9 and 46.2% for tumor volumes < 11 cc, 11–27 cc and > 27 cc, respectively (*p* = 0.0023). In a subset of patients who received BED ≥100Gy_10_, 2-year LC rates for tumor volumes < 11 cc, 11–27 cc and > 27 cc were 82.8, 58.9 and 68.6%, respectively (*p* = 0.0244), and 2-year OS rates were 66.0, 58.8 and 28.5%, respectively (*p* = 0.0081).

**Conclusion:**

Excellent OS and LC is achievable with SBRT utilizing BED ≥100Gy_10_ for lung metastases according to the RSSearch® Registry data. Patients with small lung metastases (volumes < 11 cc) had better LC and OS when using SBRT doses of BED ≥100Gy_10_. Further studies to evaluate a difference, if any, between various tumor types will require a larger number of patients.

## Introduction

Pulmonary metastases are a very frequent occurrence in patients with cancer. One series of a thousand patients found that 50% who suffered a malignancy-related death had the presence of pulmonary metastases at the time of autopsy [1]. A large surgical series of cancer patients with lung metastases treated with metastasectomy revealed a 15-year survival of 22%, an unexpected outcome for patients with stage 4 disease [2]. Researchers have found genomic differences in microRNA expression of these limited metastatic tumors compared to their widely metastatic counterparts, lending credence to the idea that our binary system of local or metastatic disease might be incorrect [3, 4]. Hellman et al. coined a limited metastatic state titled oligometastases where aggressive surgical and ablative therapies could potentially lead to long disease free intervals [5].

Metastasectomy for lung metastases has been the standard of care but is often not possible due to medical comorbidities, extrathoracic disease, unresectable metastases, or short disease free intervals. An ablative therapy such as stereotactic body radiation therapy (SBRT) has been reported in many retrospective reports for lung metastases however, with limited sample size [6]. We present a large series of metastatic lung tumors treated with SBRT using the RSSearch® Registry.

## Materials and methods

The RSSearch® Registry is an international, web-based registry designed for SBRT and stereotactic radiosurgery (SRS) research with currently over 18,000 patients enrolled (www.clinicaltrials.gov/NCT01885299) [7]. RSSearch® was designed to standardize the collection of patient screening, treatment and outcome data for patients treated with SBRT and SRS with the goal of conducting research outcomes analysis to identify the most effective and appropriate clinical uses of SRS/SBRT. RSSearch® is managed by the Radiosurgery Society, a non-profit, professional medical society (www.therss.org) and adheres to the Health Insurance Portability and Accountability Act (HIPAA) in all domains including database security, data transmission, and confidentiality. The database is contracted and maintained by Advertek (Nashville, TN). An audit was performed by the study investigators of sites participating in this study which outlined missing data points. Centers were asked to provide missing data which was generally successful in recapturing this data; however we are unable to quantify its success rate.

Data collected in RSSearch® includes the following categories: patient demographics, treated lesion (size, volume, location), treatment plan including use of surgery or chemotherapy, information on SBRT delivery including dose and fractionation, toxicity, symptom control, lesion response, survival data, and progression data. Aggregate de-identified data is accessible by RSSearch® administrators. Requests for retrospective data analysis are sent to the RSSearch® Review Committee, which approves or denies all requests for data.

Lesion locations and SBRT treatment sites are described using the World Health Organization (WHO) International Classification of Diseases (ICD), version 9 codes. Toxicity data is coded using the Common Toxicity Criteria for Adverse Event Reporting, version 3. The majority of patients were treated with the CyberKnife™ Robotic Radiosurgery System (Accuray Inc., Sunnyvale, CA) and 2 patients were treated with Truebeam (Varian Medical Systems, Palo Alto, CA). Due to the nature of the current study using registry data, no pre-defined treatment planning criteria were enforced and instead relied upon individual institutional guidelines. After consensus review of the physicians from the treatment sites, the majority of patients were simulated in the supine position using computed tomography (CT) scanning above and below the region of interest during inspiration, expiration, and free breathing. One millimeter slice reconstruction of the treatment planning area was transferred to the treatment planning station. Positron emission tomography (PET) scans were used to aid in target volume delineation via image fusion to the CT scan. Target volumes were delineated by physician (radiation oncologist, pulmonologist, or surgeon) using CT and PET scans. Gross tumor volume (GTV) was often used as the clinical target volume (CTV), with a 3–10 mm margin added circumferentially to define the planning target volume (PTV).

Real time tumor tracking was incorporated for patients treated with CyberKnife using the Synchrony® Respiratory Motion Tracking System. Radiation dosimetry on patients treated on the CyberKnife system was planned using the MultiPlan® System (Accuray Incorporated, Sunnyvale, CA) which incorporated non-isocentric and non-coplanar radiation delivery using Monte Carlo or Ray Tracing algorithms. Ray Tracing generally overestimates tumor dose in the lung due to lack of capacity to account for the lung-tumor density heterogeneity. Therefore Ray Tracing dose was converted into a more accurate Monte Carlo equivalent dose using an equation based on the tumor size. Patients who did not have tumor size information available within the RSSearch® Registry were excluded from BED analysis (*n* = 20). In comparing various fractionation schema and doses, biologically effective dose (BED Gy_10_) was calculated using the linear quadratic model.

All centers performing SBRT/SRS are able to participate in RSSearch®. No compensation is given to patient participants or participating centers. Institutional Review Board (IRB) approval is required at each participating center, and patients must give informed consent. Data is entered into RSSearch® usually in a prospective fashion however retrospective data entry is allowed and coded as such.

Patient follow-up was performed per institutional guidelines and the date of last follow up used for actuarial analysis with all time intervals considered. Patients were censored for survival at time of death and for local control at time of local failure. All participating centers reported follow-up clinical and imaging data. Local progression was evaluated independently for each lesion at the participating institution following a modified RECIST (Response Evaluation and Criteria in Solid Tumors) criteria which defined local progression as at least a 20% increase in the size of lesions and/or appearance of one or more lesions in target treatment location and local control was defined as disappearance of, decrease in, or no increase in size of the treated lesions.

Statistical analysis and Kaplan-Meier survival curves were performed using GraphPad and Instat Software, La Jolla, CA. Overall survival was calculated for each patient using the first date of SBRT to date of death or date of last follow up. Specific cause of death was not reported for all patients in RSSearch® and therefore not evaluated in this study. Local failure was determined for each treated tumor using last date of SBRT to date of physician reported failure. Subgroups were compared using *X* ^2^, log-rank tests and Gehan-Breslow Wilcoxon tests. Values of *p* < 0.05 were considered statistically significant.

For more information on the background on the RSSearch® Registry, please see the previous descriptive paper on its creation and use [7, 8].

## Results

### Patient characteristics

Between April 2004 and July 2015, 702 patients with lung metastases from 28 centers in the US, one center from Germany and one center from Australia, were identified in the RSSearch®Patient Registry. Of those patients, 577 had dose and fractionation information available. One hundred thirty patients were excluded from the study because of previous surgery, SBRT, or radiofrequency ablation (RFA) to the SBRT-treated area. This resulted in 447 patients with lung metastases treated with SBRT evaluable for survival. We are unable to say with certainty that these patients had no other metastatic sites due to the nature of our data collection. There were 304 of these patients with 327 lesions evaluable for LC. Median age of the group was 69 years (range 18–93). Additional patient demographics and characteristics are found in Table 1.

**Table 1:** Patient characteristics and SBRT treatment details

Variable	N (%)
Median Age, years (range)	69 (18–93)
Gender	
Male	223 (49.9%)
Female	221 (49.4%)
Not reported	3 (0.01%)
Median Karnofsky Performance Score (range)	90 (30–100)
Ethnicity	
African-American	24 (5.4%)
Asian	2 (0.5%)
Caucasian	325 (72.7%)
Hispanic	17 (3.8%)
Other	1 (0.2%)
Unknown	14 (3.1%)
Not reported	64 (14.3%)
Prior Chemotherapy	220 (49.2%)
Median Lesion Volume, cc (range)	10.58 (0.1–654.5)
Primary Tumor Location	
Breast	41 (9.2%)
Colorectal	115 (25.7%)
Head & Neck	51 (11.4%)
Lung	74 (16.6%)
Kidney	36 (8.1%)
Skin	29 (6.5%)
Other	99 (22.1%)
Not reported	2 (0.4%)
Median SBRT Dose, Gy (range)	50 (8–60)
Median Max Dose, Gy (range)	68.2 (10.3–78)
Median Number of Fractions (range)	3 (1–8)
Median Monte Carlo Dose, Gy (range)	45.6 (8–60)
Median Monte Carlo Dose (Gy) per Fraction	13 (2–40)
Median Monte Carlo BED, (range)	100 (9–204)

### Tumor characteristics

Primary tumor types included colorectal (CRC) (25.7%), lung (16.6%), head and neck (H&N) (11.4%), breast (9.2%), skin (6.5%), gynecologic (6%), sarcoma (4%), bladder/ureter (4%), and other (16%). Median Karnofsky Performance Status (KPS) was 90 (range 25%/75% 80–100). Median follow up was 13 months (range 25%/75% 6–26 months, max 123 months). Median number of metastases was 1 (range 1–3). Median metastasis size was 10.58 cc (range 0.1–654.5 cc).

### Treatment characteristics

Median dose of SBRT to target metastasis was 50 Gy (range 25%/75% 48–54 Gy). The median corrected Monte Carlo dose was 45.6 Gy (range 25%/75% 40.6–50.7 Gy). The median bioequivalent dose was 100Gy_10_ (range 25%/75% 81–136). Median number of fractions was 3 (range 25%/75% 3–5). The median Monte Carlo dose per fraction was 13 Gy/fraction (range 25%/75% 10–17 Gy/fraction).

### Outcome data

**Fig. 1:**
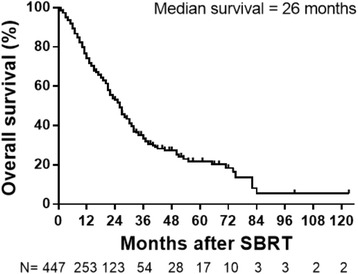
Median OS for the entire group was 26 months after SBRT. Tick marks indicate censored patients. Number of patients for each time point shown below graph

**Fig. 2:**
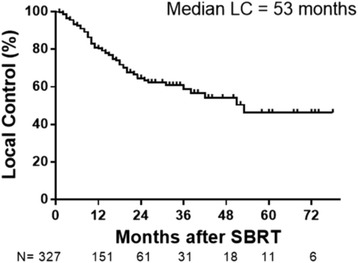
Median LC for the entire group was 53 months after SBRT. Tick marks indicate censored patients

Median overall survival (OS) for the entire group was 26 months. The 1-, 3- and 5- year OS were 74.1, 33.3, and 21.8% of patients, respectively (Fig. 1). Median LC for the entire group was 53 months. The 1-, 3-, and 5-year LC rates were 80.4, 58.9, and 46.2%, respectively (Fig. 2).

**Fig. 3:**
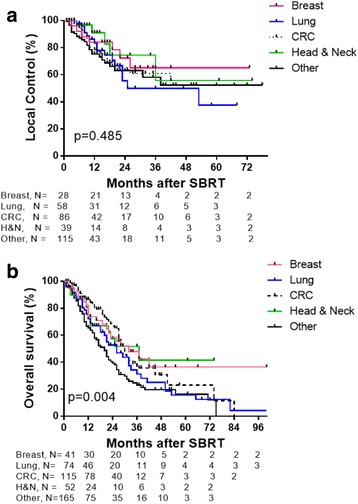
Kaplan-Meier graphs showing LC (**a**) and OS (**b**) for patients treated with SBRT and stratified by primary tumor type (breast (*pink*), lung (*blue*), CRC (*hashed black*), head and neck (*green*) and other (*solid black*). Number of patients are shown. *Tick marks* indicate censored patients

We investigated whether primary tumor type (breast, lung, colorectal cancer (CRC), head and neck or other) had an effect on LC and OS following SBRT treatment. There was no statistical difference in LC rates (*p* = 0.485 by log-rank test; *p* = 0.181 by Gehan-Breslow Wilcoxon test, Fig. 3a) for the primary tumor types. The 2-year LC rates for breast, lung, CRC, head and neck, and other primaries were 72.4, 55.6, 65.4, 74.4 and 63.1%, respectively. A statistical difference in OS rates was observed for primary tumor types, with head and neck patients having a median OS of 37 months, breast cancer patients had a median OS of 32 months, CRC patients had a median OS of 30 months, lung cancer patients had a median OS of 26 months, and all other primary tumors had a median OS of 20 months (*p* = 0.004 by log-rank test; *p* = 0.0024 by Gehan-Breslow Wilcoxon test, Fig. 3b). The 3-year OS rates for breast, CRC, lung, head and neck, and other primary tumors were 47.9, 31.3, 35.8, 51.8, and 23.1%, respectively.

**Fig. 4:**
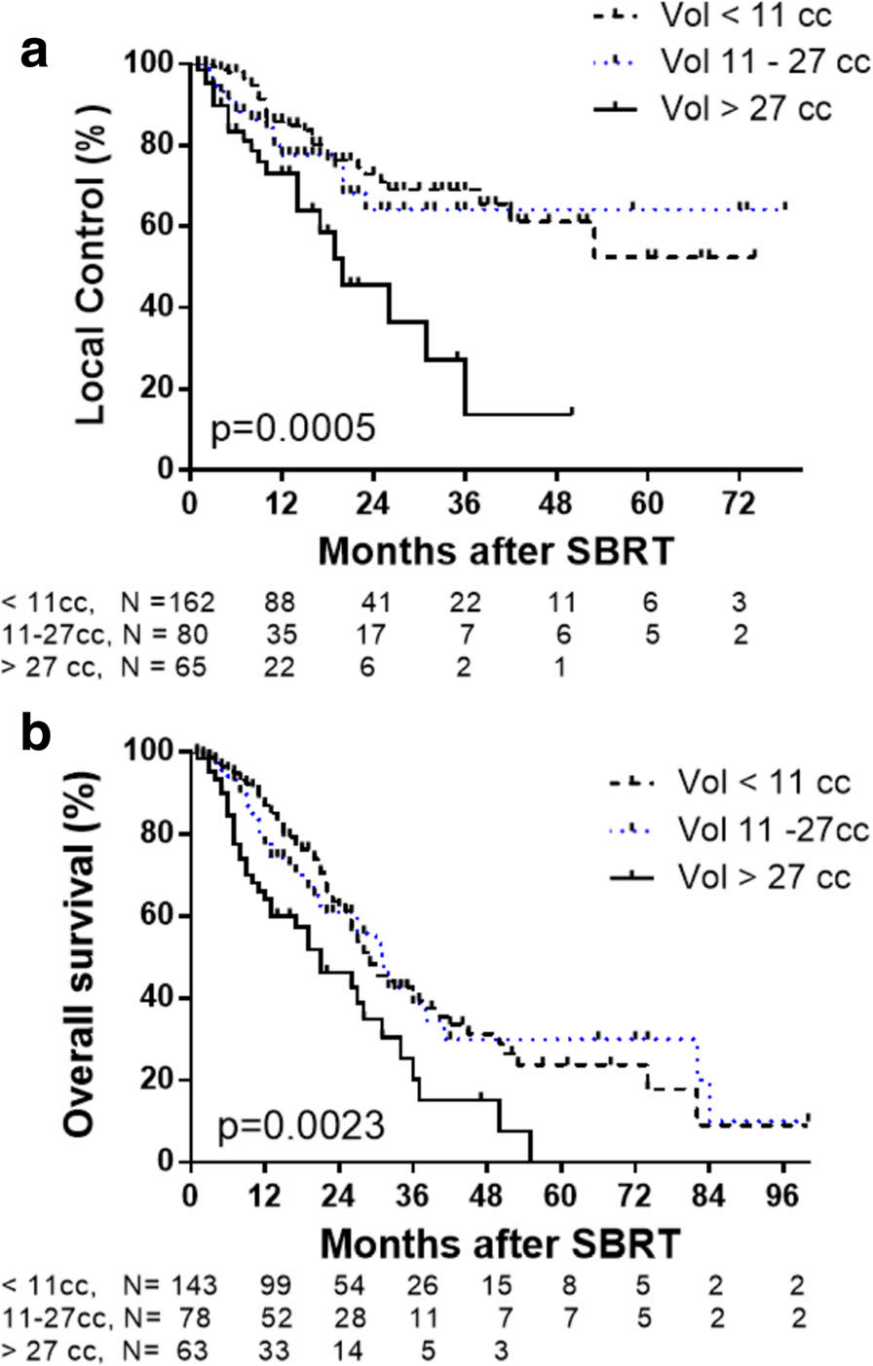
Kaplan-Meier graphs showing LC (**a**) and OS (**b**) for patients treated with SBRT and stratified by tumor volume < 11 cc (*black hashed line*), 11–27 cc (*blue dotted line*), and >27 cc (*solid black line*). Tick marks indicate censored patients

We next investigated whether lung metastases tumor volume was associated with LC and/or OS. Because median and average metastasis volumes were 10.58 cc and 26.72 cc, respectively, lesions were stratified into three groups: tumor volume <11 cc, 11–27 cc, and >27 cc. A statistical difference was noted between the three groups, with improved LC for smaller tumors. Two-year LC was 72.9, 64.2 and 45.6% for tumor volumes < 11 cc, 11–27 cc and > 27 cc, respectively (*p* = 0.0005 by log-rank test; *p* = 0.0011 by Gehan-Breslow Wilcoxon test Fig. 4a). This translated into improved OS, with 2-year OS of 62.4, 60.9, 46.1% for tumor volumes < 11 cc, 11–27 cc, and > 27 cc, respectively, and median OS for lesions <11 cc, 11–27 cc, and >27 cc was 29, 31, and 21 months respectively (*p* = 0.0023 by log-rank test; *p* = 0.0011 by Gehan-Breslow Wilcoxon test, Fig. 4b).

**Fig. 5:**
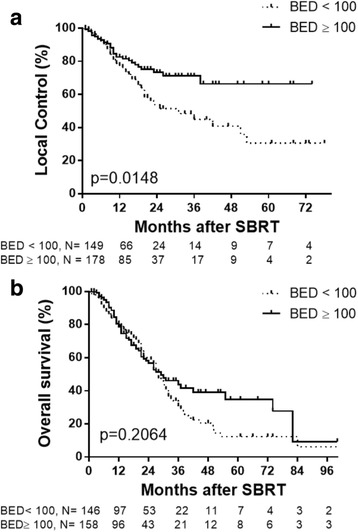
Kaplan-Meier graphs of LC (**a**) and OS (**b**) for patients treated with SBRT stratified by BED <100Gy_10_ (*dotted line*) and BED ≥ 100 Gy_10_(*solid black line*). *Tick marks* indicate censored patients

All patients were stratified by BED < 100Gy_10_ and BED ≥100Gy_10_ and Kaplan-Meier analysis was used to assess LC and OS. Lung metastases treated with BED ≥100Gy_10_ had improved LC compared to lesions that received BED ≤ 100Gy_10_ (Fig. 5a). LC rates at 1-, 3-, and 5-years were 82.6, 71.1, and 66.4%, respectively, for lesions treated with BED ≥ 100Gy_10_, and 77.6, 44.9 and 30.7%, respectively, for lesions treated with BED < 100Gy_10_ (*p* = 0.0148 by log-rank test). This did not translate into a significant difference in OS (*p* = 0.2064) (Fig. 5b).

**Fig. 6:**
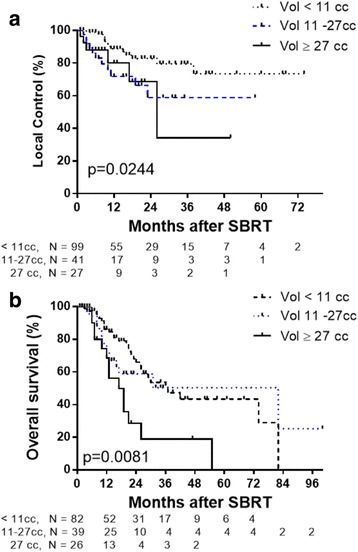
Kaplan-Meier graphs of LC (**a**) and OS (**b**) of lung metastases treated with SBRT BED ≥100Gy_10_ stratified by volume of lesion < 11 cc (*dotted line*), 11–27 cc (*blue line*), and ≥11 cc (*solid line*). Tick marks indicate censored patients

Stratification was then performed using only lung metastases treated with BED ≥100Gy_10_ stratified by lesion volume <11 cc, 11–27 cc, and ≥27 cc. Two-year LC rates for tumors < 11 cc, 11–27 cc and > 27 cc were 82.8% and 58.9% and 68.6%, respectively (*p* = 0.0244 by log-rank test; *p* = 0.0122 by Gehan-Breslow Wilcoxon test, Fig. 6a). Two-year OS for patients with tumors < 11 cc, 11–27 cc and 27 cc were 66.0 and 58.8% and 28.5%, respectively (*p* = 0.008 by log-rank test; *p* = 0.0133 by Gehan-Breslow Wilcoxon test, Fig. 6b).

Other factors that were evaluated during statistical analysis included age, KPS, gender, and use of chemotherapy, which were all found to be not statistically significant for LC or OS.

## Discussion

This RSSearch® Patient Registry analysis represents a report of a large cohort of patients treated with SBRT for lung metastases (*n* = 447 patients). In comparison, another large study includes 217 patients from a single-institutional prospective series and 95 patients analyzed in a single-institution retrospective study [6, 9, 10]. A meta-analysis reported by Ashworth et al. included 757 patients all with lung cancer; however this study only included 88 patients treated with SBRT and included metastases to all body sites and not only lung metastases [11]. The current study includes a heterogeneous population of patients including large sample sizes of various primary sites, tumor sizes, doses, and patient populations from across the United States, Germany and Australia.

The use of SBRT for lung metastases allows for high ablative doses of radiation with the potential for extended local control and survival as shown in the current analysis. Multiple studies since have found LC rates of isolated or few lung metastases to be 70–100% at 1 year [12–15]. Our LC rate compares favorably at 80.37%. The two-year weighted OS rate compiled in an article review by Alongi et al. was 54% (range 39–84%) [16]. In comparison, the 2-year OS rate for all patients reported in our study compares similarly at 53.02%.

In assessing LC, most studies support using a BED of at least 100Gy_10_ to have LC comparable to metastasectomy of pulmonary metastases [17, 18]. Doses of less than 100 Gy_10_ however are still used in clinical practice with high LC rates and minimal toxicity [13]. In our current study, we saw a statistically significant difference with higher LC rates in the BED ≥ 100Gy_10_ group, adding further evidence for its use. This LC rate did not translate into improved OS for all size lesions, but when stratifying by metastasis volume there was a trend for improved OS in lesions smaller than 11 cc.

In the current study, we saw differences in OS between primary histology types, favoring improved OS for H&N, breast, and CRC but without difference in local control by primary histology. Takeda et al. found CRC lung oligometastases to have poorer LC than other histologies (lung, H&N) [10]. These results do not corroborate with our results, as oligometastatic tumors from lung primaries fared worse in the present study compared to Takeda et al. where other histologies (including lung primary oligometastatic tumors) fared better. Takeda et al. was limited to a small sample size of patients with lung oligometastases (*n* = 44) of which half were from colorectal cancer compared with our study with a much larger cohort. In addition, other studies have found no relationship between oligometastatic lung tumors from CRC primary vs other primary oligometastatic lung tumors on multivariate analysis (MVA) [14, 19]. Our study also compares oligometastatic lung tumors by primary individually instead of grouping primaries together for comparison.

Our study also found differences in LC and OS rates based on tumor volume, with significantly higher LC and longer OS for smaller volumes. McCammon et al. had similar findings on univariate (UVA) and multivariate (MVA) analysis, finding significant differences in rates of LC comparing values above and below their median tumor volume (8.9 cc), but this study did not see differences in OS [19]. In comparison, two previous studies treated large volume metastases with SBRT, both averaging a median volume of 41 cc per lesion treated, and found a LC rate comparable to small volume lesions, again without reporting OS [20, 21].

Approximately 25% of patients undergoing metastasectomy have long term survival [2, 22, 23]. The remaining patients usually see progression of their disease and development of new metastases within 6 months of ablative therapy [24, 25]. A greater time interval between locoregional disease and metastatic disease portends better prognosis and longer disease-free survival [2, 22]. Other factors portending to longer disease-free survival include having fewer metastases, non-synchronous metastases, stable disease before ablative therapies, estrogen-positive receptor breast cancer primaries, and complete ablations [24–26]. Because this represents a registry for patients treated with SBRT for lung metastases, many of these parameters could not be evaluated.

It is difficult to properly evaluate OS using SBRT for lung metastases in context of comparing it to metastasectomy. There is an absence of phase III randomized controlled trials, and the phase I/II trials that have been completed have patients with widely variable clinical characteristics [16]. Only 1 retrospective study by Yu et al. compared SBRT to metastasectomy in 58 patients with osteosarcoma, with OS at 40% in both groups [27]. In addition, there is a bias in selection of patients for SBRT - they are generally judged to be inoperable due to their medical comorbidities which could significantly affect OS rates [16]. We did not find significant survival differences by KPS however our dataset lacks more rigorous comorbidity scores such as the Charlson/Deyo score needed to provide survival for potentially operable patients [28].

There are weaknesses to the current study including the short median follow-up of 13 months however this follow-up is comparable to other single institution series [29, 30]. Our study includes no predefined treatment planning criteria, defined individually by the participating centers, with variability in dose and fractionation. This variability however allowed for dose response analysis due to wide ranges in dose but may have lowered the local control and survival rates possible if more uniform high dose was utilized in all patients. Our study also has the standard limitations intrinsic to registry studies: allocation of patients is not random and data collection is less robust than randomized clinical trials.

There are ongoing prospective trials that will hopefully answer if a survival benefit is found for patients treated with SBRT for oligometastases. Only one prospective trial from MD Anderson reported in abstract form found a median PFS advantage with three or less sites of oligometastases treated with SBRT, conventional external beam radiation or surgery for non-small cell lung cancer from 3.9 to 11.9 months (*p* = 0.005) [31]. In addition, other prospective trials continue to accrue including the SABR-COMET, NRG BR002, and the UK CORE trials. Until results of those trials are reported, we will have to rely on prospective registry series like the current study to guide treatment decisions.

## Conclusions

SBRT provides extended survival in patients with lung metastases, with the current study providing a 5-year actuarial survival of 21.8%. The 1-, 3-, and 5-year LC rates were 80.4%, 58.9%, and 46.2%, respectively. Smaller tumor size and the primary tumor type (H&N/colon/breast) were associated with prolonged survival. High dose BED (≥100Gy_10_) and smaller tumor size were associated with prolonged local control.
